# Sex Differences in Cannabis Use and Effects: A Cross-Sectional Survey of Cannabis Users

**DOI:** 10.1089/can.2016.0010

**Published:** 2016-07-01

**Authors:** Carrie Cuttler, Laurie K. Mischley, Michelle Sexton

**Affiliations:** ^1^ Department of Psychology, Washington State University, Pullman, Washington.; ^2^ Bastyr University Research Institute, Seattle, Washington.; ^3^ Center for the Study of Cannabis and Social Policy, Seattle, Washington.

**Keywords:** cannabis, cannabis acute effects, cannabis withdrawal, medical cannabis, sex differences

## Abstract

**Introduction:** Despite known sex differences in the endocannabinoid system of animals, little attention has been paid to sex differences in human's cannabis use patterns and effects. The purpose of the present study was to examine sex differences in cannabis use patterns and effects in a large sample of recreational and medical cannabis users.

**Methods:** A large sample (*n*=2374) of cannabis users completed an anonymous, online survey that assessed their cannabis use practices and experiences, including the short-term acute effects of cannabis and withdrawal effects. A subsample of 1418 medical cannabis users further indicated the medical conditions for which they use cannabis and its perceived efficacy.

**Results:** The results indicated that men reported using cannabis more frequently and in higher quantities than did women. Men were more likely to report using joints/blunts, vaporizers, and concentrates, while women were more likely to report using pipes and oral administration. Men were more likely than women to report increased appetite, improved memory, enthusiasm, altered time perception, and increased musicality when high, while women were more likely than men to report loss of appetite and desire to clean when high. Men were more likely than women to report insomnia and vivid dreams during periods of withdrawal, while women were more likely than men to report nausea and anxiety as withdrawal symptoms. Sex differences in the conditions for which medical cannabis is used, and its efficacy, were trivial.

**Conclusions:** These results may be used to focus research on biological and psychosocial mechanisms underlying cannabis-related sex differences, to inform clinicians treating individuals with cannabis use disorders, and to inform cannabis consumers, clinicians, and policymakers about the risks and benefits of cannabis for both sexes.

## Introduction

The rapid growth of the cannabis industry, changes in public perceptions about the risks of cannabis,^1^ and increasing rates of cannabis use^2^ make it imperative that research is conducted to better understand the effects of cannabis in humans. This information will enable the expanding population of cannabis consumers, as well as healthcare professionals and policymakers, to make informed decisions about the risks and benefits of cannabis use. To fully understand the effects of cannabis, potential sex differences must be considered. Animal research on the endocannabinoid system has revealed a sexually dimorphic system that interacts with gonadal hormones.^3–5^ Correspondingly, the small literature on sex differences in humans hints at intriguing differences in men's and women's experiences with cannabis.

### Cannabis use, dependence, and treatment

The most well documented sex difference in human cannabis users is that men are more likely to use and become dependent on cannabis.^6–16^ Men are also more likely to use cannabis for medicinal purposes^17^ and initiate use at a younger age.^18–20^ While there is some evidence that the “gender gap” in cannabis use is decreasing^17,21,22^ existing sex differences in cannabis use may reflect women's increased perceptions of risks associated with regular use.^1,23,24^ Further, while men are more likely to become dependent on cannabis, women demonstrate a more rapid progression from first use to cannabis use disorder.^25–30^ Consistent with these results, young males are more likely to enter treatment for cannabis abuse^31^ but women tend to enter treatment for cannabis abuse after fewer years of use and less cumulative use than men.^26^

### Withdrawal

Research findings on sex differences in self-reported symptoms of cannabis withdrawal are equivocal: some studies suggest that women report experiencing more withdrawal symptoms than men,^32,33^ and others fail to find sex differences in withdrawal symptoms.^10,25,34,35^ Some of the studies that failed to detect significant sex differences in withdrawal symptoms reported that the men in their samples reported greater use and dependence on cannabis.^10,25^ Therefore, it may be that women's decreased cannabis use and dependence helps to explain the lack of significant sex differences in withdrawal symptoms in those studies.

Agrawal et al. also failed to find sex differences in the overall prevalence of withdrawal symptoms; however, they did find that women were more likely to report nausea, and men were more likely to report goose bumps and pupil dilation, as withdrawal symptoms.^35^ Herrmann et al. also found that women reported more nausea, as well as stomach pain, irritability, restlessness, anger, and outbursts during their last quit attempt than did men.^32^ Finally, Copersino and colleagues found that women were more likely to report upset stomach whereas men were more likely to report cannabis cravings and increased sex drive during cannabis withdrawal.^36^ Therefore, while it is unclear whether women experience worse withdrawal than men, evidence indicates that women are prone to experience different withdrawal symptoms, particularly nausea and upset stomach.

### Acute effects

Research involving the administration of cannabis or delta-9-tetrahydrocannabinol (THC) suggests that women report significantly more dizziness than men.^37^ Findings on the subjective effects of cannabis or THC intoxication are equivocal: one study found that women give higher ratings than men for the subjective effects of smoked cannabis,^38^ one found that men report greater subjective effects of oral THC (2.5, 5, 10 mg) than women do,^39^ and one indicated that sex differences in subjective effects of oral THC vary as a function of dose, with women reporting greater subjective effects than men at low doses (5 mg) and men reporting greater subjective effects than women at high doses (15 mg).^40^ Finally, Penetar et al. found shorter latencies to detect the effects of smoked cannabis and longer duration of the effects of smoked cannabis in men compared to women.^41^ These inconsistent results may reflect differences in in the populations tested (regular cannabis users^37,38,40,41^ vs. non-users^39^), the route of administration used (smoked cannabis^37,38,41^ vs. orally administered THC^37,39,40^), and sex differences in frequency and quantity of cannabis used (only one study matched men and women on frequency and quantity of use^38^). Clearly additional research is needed before strong conclusions regarding sex differences in the subjective effects of cannabis can be reached.

### Cognition

To our knowledge only one sex difference in the acute effects of cannabis on cognition has been reported. Surprisingly, this study reported that a low dose of sublingually administered THC improved the spatial working memory performance of female but not male cannabis users.^42^ The remaining literature on sex differences in the influence of cannabis on cognition has focused on the chronic or residual effects and has once again produced somewhat equivocal results. For instance, Pope et al. are often cited for finding that female heavy cannabis users demonstrate worse visuospatial memory test performance relative to female light cannabis users (no differences were found in men)^43^; however, the same researchers previously reported the opposite result using a different test of visuospatial memory.^44^ Crane et al. reported that cannabis use was associated with poorer decision-making in men but not women.^45^ Subsequently, Crane et al. reported that cannabis use was associated with better decision-making in women but not men.^46^ These researchers also found that cannabis use was associated with poorer episodic memory in women but not men.^45,46^ More consistently, male, but not female, cannabis users have demonstrated decreased speed than non-users on timed cognitive tests.^43,44,47,48^ Therefore, while converging evidence suggests that chronic cannabis use may diminish speed of performance in men but not women, evidence from other domains of cognition is equivocal and future research is needed to better understand potential sex differences in the influence of cannabis on different cognitive domains.

### Study purpose

While previous research suggests that men and women may use cannabis differently and experience different effects, many of the results pertaining to sex differences are equivocal. The primary purpose of the present survey study was to further document and describe sex differences in self-reported cannabis use and effects using a large sample of recreational and medical cannabis users. Moreover, we sought to explore potential sex differences in the medical conditions that medical cannabis patients report using cannabis to treat as well as in the perceived efficacy of cannabis in the treatment of those conditions. To our knowledge this is the first study to examine sex differences in medical cannabis use and effects.

## Methods

### Participants

A self-selected convenience sample of 2459 participants was recruited via word-of-mouth and links on advertisements posted on various websites and in Washington State cannabis dispensaries. The only inclusion criterion was use of cannabis in the past 90 days. Twenty-five respondents did not meet that criterion and were excluded. Thirty respondents were identified as providing more than one set of responses (based on redundant unique self-generated identification codes, age, and gender data) and the data from their second set of responses were excluded. An additional 30 respondents did not indicate their gender and were therefore excluded. The final sample included 2374 (1370 male, 1004 female) cannabis users. A total of 70.1% identified as recreational users, 59.7% as medical users, 2.4% as religious users (i.e., use of cannabis for primarily religious ceremonies and/or spiritual purposes), and 32.2% as both recreational and medical users. The sample was well balanced with respect to sex but was predominantly Caucasian. Table 1 shows the complete demographic characteristics of the sample broken down by sex.

**Table 1.** **Demographic Characteristics of Male and Female Cannabis Users**

	Male	Female	*p*
Gender (*n*)	1370	1004	
	57.7%	43.3%	
Age (*n*)	1354	990	
	*M*=32.73 (*SD*=13.20)	*M*=34.71 (*SD*=13.12)	**<0.001**
Ethnicity (*n*)	1350	997	
Caucasian	87.5%	85.9%	0.25
African American	1.2%	1.2%	0.97
Hispanic	2.9%	3.8%	0.22
Native American	0.7%	1.8%	0.011
Asian/Pacific Islander	1.6%	1.5%	0.92
Other	6.2%	5.8%	0.68
Highest level of education (*n*)	1356	999	
<High school	4.1%	2.1%	**0.008**
High school/GED	33%	24.3%	**<0.001**
Technical school	12.3%	10.1%	0.10
Associate	13.3%	16.6%	0.02
Bachelors	26.9%	31.8%	**0.009**
Masters	6.6%	9.9%	**0.004**
Doctorate	3.8%	5.1%	0.14
Current employment (*n*)	1361	994	
Full time	54.7%	48.4%	**0.003**
Part time	20.4%	23.6%	0.06
Unemployed	13.7%	13.5%	0.90
Retired	4%	3.5%	0.52
Disabled	7.3%	11%	**0.002**
Income: last 12 months (*n*)	1328	974	
<$20,000	19.7%	24.2%	**0.009**
$20,000–40,000	22.5%	26.2%	0.04
$40,000–60,000	16.3%	18.6%	0.15
$60,000–80,000	11.2%	9.8%	0.26
$80,000–100,000	10.5%	8.1%	0.06
$100,000–150,000	11.1%	7%	**<0.001**
>$150,000	8.7%	6.2%	0.03
Relationship status (*n*)	1367	998	
Married	29.3%	32.6%	0.09
Domestic partnership	12.4%	16.9%	**0.002**
Divorced	3.8%	6.6%	**0.002**
Single	47.1%	36.6%	**<0.001**
Other	7.3%	7.3%	1.0

Bold values indicate statistical significance.

SD, standard deviation.

### Procedure

A brief, anonymous, online survey was used to assess cannabis use patterns and effects. No compensation was provided to participants. The survey was developed by examining strengths and weaknesses of existing surveys, previous literature, DSM criteria for cannabis withdrawal, medical indications for cannabis use, and clinical experience. Drafts were circulated to physicians, researchers, and cannabis users for feedback in an iterative process. Bastyr University's Internal Review Board approved the protocol.

Some aspects of the collected data are not reported here because they are being published separately. Specifically, data on the medical conditions and perceived efficacy of cannabis in treating medical conditions (disregarding sex) and data on driving beliefs and habits are presented in two separate manuscripts that are currently under review. Data on the use of cannabis as a substitute for alcohol and other drugs are currently in preparation for a separate manuscript. Only questions relevant to the present study are described in the Materials section that follows.

### Materials

#### Demographic questions

Participants were asked to input their age and country of residence. Participants were asked to use nominal scales to indicate their gender, ethnicity, employment status, and relationship status. Finally, they used ordinal scales to indicate their highest level of education and total family income. The response options for these questions are shown in Table 1.

#### Cannabis use patterns

Participants were asked to use a nominal scale to indicate whether or not (yes/no) they used cannabis for recreation purposes, medicinal purposes, and religious purposes. They were asked to use a nominal scale to indicate the method of administration they most commonly use. They were also asked to use ordinal scales to indicate frequency of cannabis use, quantity of cannabis used per week, and age of first use. Complete response options for each of these items are shown in Table 2. Finally, participants indicated the number of hits they take per smoking session, using a ratio scale ranging from 1 (1 hit) to 11 (more than 10 hits).

**Table 2.** **Cannabis Use Patterns of Male and Female Cannabis Users**

	Male	Female	*p*
Frequency of use (*n*)	1364	1004	
All day, everyday	10.4%	7.2%	**0.007**
5–10 times per day	14.1%	10.3%	**0.005**
1–4 times per day	42.7%	43.3%	0.78
3–6 times per week	14.6%	14.5%	0.97
1–3 times per week	10.3%	10.7%	0.80
2–3 times per month	4.0%	6.0%	0.02
Once a month	1.2%	2.7%	**0.006**
Less than once a month	2.6%	5.4%	**<0.001**
Quantity (per week) (*n*)	1359	992	
More than 1 ounce (28 g)	1.9%	2.4%	0.40
1 ounce (28 g)	5.4%	3.3%	0.015
1/4 ounce (7 g)	25.5%	16.7%	**<0.001**
3–5 g	29.7%	30.9%	0.50
1–2 g	21.7%	21.8%	0.97
Less than 1 g	15.7%	24.8%	**<0.001**
Hits per session (*n*)	1364	975	
	*M*=6.08 (*SD*=3.39)	*M*=5.00 (*SD*=2.89)	**<0.001**
Age of first use, years (*n)*	1369	1000	
<14	14.2%	15.4%	0.40
14–16	38.3%	38%	0.89
17–18	25.1%	20.8%	0.02
19–20	9.8%	10.3%	0.68
21–25	7.7%	9.1%	0.21
26–30	3.0%	2.4%	0.38
>30	2.1%	4%	**0.005**
Method of use (*n*)	1366	1003	
Joints/blunts	23%	16.5%	**<0.001**
Pipe	28.4%	42.3%	**<0.001**
Bong	21.1%	17.8%	0.05
Vaporizer	17.3%	11.4%	**<0.001**
Oral (edibles, tinctures, capsules)	3.9%	7.9%	**<0.001**
Concentrates	5.4%	3.1%	**0.007**
Topical	0.4%	0.4%	1.0
Other	0.6%	0.7%	0.73

Bold values indicate statistical significance.

#### Addiction and withdrawal

Participants used a nominal (yes/no) scale to indicate whether they had ever had trouble reducing or stopping their use of cannabis, and they used a nominal (yes/no/I don't know) scale to indicate whether they believed that cannabis is addictive.

Participants were asked to report the withdrawal symptoms experienced with discontinuation of cannabis for 72 h or more. They were shown a list of 13 withdrawal symptoms and were asked to use a nominal (yes/no) scale to indicate which they have experienced. The list of withdrawal symptoms is shown in Table 3. A not applicable (n/a) response option was also included for those who had not experienced withdrawal or who had never discontinued cannabis for 72 h or more. Finally, a composite withdrawal score was computed by summing the number of withdrawal symptoms each participant endorsed.

**Table 3.** **Sex Differences in Addiction and Withdrawal Symptoms**

	Male, *n*=1370	Female, *n*=1004	*p*
Addictiveness			
Difficulty in reducing or stopping	17.7%	15.2%	0.11
Believe cannabis is not addictive	70.6%	67.7%	0.13
Believe cannabis is addictive	18.2%	14.8%	0.03
Do not know if cannabis is addictive	11.3%	17.5%	**<0.001**
Withdrawal symptoms			
Irritability	35.9%	32.2%	0.06
Insomnia/interrupted sleep	32%	27%	**0.009**
Nausea	5.3%	8.4%	**0.003**
Loss of appetite	20.8%	18.3%	0.14
Sweating	4.7%	3.1%	0.05
Salivation	0.7%	0.5%	0.48
Tremor	1.5%	1%	0.25
Weight loss	3.9%	3.9%	0.94
Tiredness	8.6%	8.8%	0.90
Vivid dreams	21.7%	13.3%	**<0.001**
Anxiety	20.5%	25%	**0.009**
Loss of productivity	13.1%	11.4%	0.19
Improved productivity	5.3%	3.8%	0.08

Bold values indicate statistical significance.

#### Acute effects

Participants were shown a list of 41 possible effects of cannabis and were asked to use a nominal (yes/no) scale to indicate which they experience when they are stoned. The list of acute effects is shown in Table 4.

**Table 4.** **Sex Differences in the Acute Effects of Cannabis**

	Male, *n*=1370	Female, *n*=1004	*p*
Cognitive			
More forgetful	37.8%	36.5%	0.50
Short-term memory problems	42.9%	41.7%	0.56
Long-term memory problems	4.2%	4.7%	0.54
Memory improvement	15.5%	11.8%	**0.009**
Difficulty concentrating	17%	15.8%	0.45
Improved concentration	42.9%	37.7%	0.011
Difficulty making decisions	9.7%	12.5%	0.03
Confusion	5.1%	5.1%	0.97
Sense of clarity/perspective	43.8%	45.9%	0.30
Difficulty finding words	16.8%	19.5%	0.09
More articulate or communicative	42.7%	41%	0.42
Social			
Better social interactions	49.7%	45%	0.03
Worse social interactions	11.8%	14.1%	0.09
More extraverted, “outward” focus	24.9%	24.5%	0.83
More “inward” focus	50.7%	49.1%	0.43
Psychological			
More calm or peaceful	79.6%	79.2%	0.79
Less anxious or fearful	55.3%	57.2%	0.37
Increased anxiety	8.8%	9.2%	0.73
Paranoia	14.5%	15.2%	0.59
Apathetic (lack of interest/concern)	9.9%	8.9%	0.38
Loss of motivation	23.6%	24.8%	0.48
Increased motivation	47%	47.6%	0.77
Enthusiastic	41.8%	34.5%	**<0.001**
Altered sense of time	41%	34.2%	**0.001**
Hallucinations	4%	3.4%	0.38
Movement			
Poor balance (feel unsteady)	5.3%	6.2%	0.34
Lack of coordination	5.3%	5.8%	0.64
Desire to stretch/exercise	36.5%	38.9%	0.22
Desire to be still (couch-lock)	32.6%	29.1%	0.07
Desire to clean	36.4%	47%	**<0.001**
Physiological			
Tired or sleepy	43.2%	48.1%	0.02
Stimulated or energized	45.3%	42%	0.11
Dry mouth	64.7%	62.8%	0.34
Dizziness	4.7%	4.4%	0.68
Increased sex drive	51.3%	46.4%	0.02
Diminished sex drive	6.9%	5.7%	0.24
Desire to eat (munchies)	75.2%	70%	**0.005**
Loss of appetite	8.5%	11.8%	**0.009**
Artistic			
More creative	75.5%	71.3%	0.02
Less creative	3.3%	3%	0.68
Musical	49.3%	37%	**<0.001**

Bold values indicate statistical significance.

#### Medical use and perceived efficacy

Participants were shown a list of 19 medical conditions and were asked to indicate which they use cannabis to treat. The list of medical conditions is shown in Table 5.

**Table 5.** **Sex Differences in Medical Conditions Cannabis Is Used to Treat**

	Male, *n*=774	Female, *n*=644	*p*
Multiple sclerosis	0.7%	1.7%	0.06
Cancer	3.4%	3.4%	0.85
Epilepsy	0.9%	1.7%	0.18
HIV	1.2%	0.2%	0.02
Glaucoma	2.3%	1.1%	0.08
IBS	12%	18.2%	**0.001**
Colitis/Crohn's disease	3.1%	3.3%	0.86
Tremor	2.3%	2.3%	1.0
Anxiety	50.6%	66.6%	**<0.001**
Depression	48.7%	52.3%	0.17
Spasticity	16.5%	21%	0.03
Nausea	20.7%	35.2%	**<0.001**
Pain	59.3%	63.8%	0.08
Intractable pain	11%	12.3%	0.45
Tics	2.8%	2.2%	0.43
Anorexia	7.8%	12.6%	**0.002**
Seizures/spasticity	2.8%	2.3%	0.55
Headaches/migraines	27.4%	45%	**<0.001**
Arthritis	15.4%	19.3%	0.054

Bold values indicate statistical significance.

IBS, Irritable Bowel Syndrome.

To assess perceived efficacy of cannabis, participants were also asked to indicate the effect that cannabis has on their medical conditions, using a scale ranging from −5 (worsening symptoms) to 0 (no change) to +5 (improvement of symptom). Each symptom was assessed separately and an additional composite score was created by computing the mean rating across all conditions.

### Data analysis

The data were analyzed using IBM SPSS 23. Nominal scale data were analyzed by computing the percentages of men and women who endorsed each response, and sex differences in these percentages were determined using chi-square tests. Sex differences in ordinal data were determined using Mann–Whitney U tests, and sex differences on interval and ratio scale data were determined using *t*-tests.

Due to the large number of comparisons and the large sample size, only results with a *p*-value below 0.01 were considered statistically significant. Ratio and interval scale data were screened for univariate outliers, defined as scores falling more than 3.29 standard deviations from the mean, and the small number of outlying values detected (<0.5% of screened data) were replaced with the nearest non-outlying value.^49^

## Results

### Cannabis use patterns

There were significant differences in the percentages of men and women who indicated using cannabis for recreational purposes (73.4% men; 65.5% women, *p*<0.001) and who indicated using cannabis for medicinal purposes (54.3% men; 64.1% women, *p*<0.001), but there was no sex difference in the percentages of men and women who reported using cannabis for religious reasons (2.5% men; 2.3% women, *p*=0.76).

The results further indicated that men use cannabis more frequently (*p*<0.001), and in higher quantities (*p*<0.001; Table 2). Men were also found to report taking significantly more hits per smoking session than women, *t*(2316)=8.06, *p*<0.001. Overall, there was no significant sex difference in the reported age of first use (*p*=0.76); however, women were twice as likely as men to report first using cannabis after the age of 30. The results of analyses on method of administration further indicate that men were significantly more likely than women to report using joints/blunts, vaporizers, and concentrates, while women were significantly more likely than men to report using pipes and oral methods of administration (Table 2).

### Addiction and withdrawal

As shown in Table 3, there were no significant sex differences in reported trouble reducing or stopping cannabis use, in beliefs that cannabis is addictive, or in beliefs that cannabis is not addictive. However, significantly more women than men reported not knowing if cannabis is addictive.

The results of analyses on the specific withdrawal symptoms revealed that men were significantly more likely than women to report experiencing insomnia and vivid dreams, while women were significantly more likely than men to report nausea and anxiety as withdrawal symptoms (Table 3). A total of 34.4% of the sample responded using the “n/a” option, indicating that they had not experienced withdrawal and/or had never discontinued cannabis for 72 h or more. Significantly more women (39.3%) than men (31%) responded with the “n/a” option (*p*<0.001). An examination of the composite score that was computed revealed that 40.2% of the sample (36.7% of men; 44.9% of women, *p*<0.001) indicated no withdrawal symptoms; while the remaining 59.7% of participants (63.3% of men; 55.1% of women, *p*<0.001) indicated that they experienced at least one withdrawal symptom. Overall, there was no significant difference in the mean number of withdrawal symptoms reported by men (*M*=1.73) and women (*M*=1.55), *t*(2372)=2.26, *p*=0.02.

### Acute effects

The results of analyses on the acute effects data indicate that men were significantly more likely than women to report experiencing memory improvement, a desire to eat, increased enthusiasm, altered sense of time, and increased musicality; in contrast women were significantly more likely than men to report experiencing a loss of appetite and desire to clean (Table 4).

### Medical use and perceived efficacy

Only the 1418 participants who indicated that they use cannabis for medicinal purposes were included in the analyses of medical use and perceived efficacy. As shown in Table 5, women were significantly more likely than men to report using cannabis to treat anxiety, nausea, anorexia, irritable bowel syndrome (IBS), and headaches/migraines.

As shown in Table 6, men reported experiencing greater headache/migraine relief from medical cannabis than women. An analysis of the composite score representing the mean rating across all conditions confirmed no significant differences in men's (*M*=3.61) and women's (*M*=3.58) ratings of the perceived efficacy of medical cannabis, *t*(1319)=0.58, *p*=0.56.

**Table 6.** **Sex Differences in Perceived Efficacy of Cannabis to Treat Various Medical Conditions**

	Male, *n*=774	Female, *n*=644	*p*
Multiple sclerosis	*n*=5; *M*=3.96 (*SD*=0.96)	*n*=11; *M*=3.28 (*SD*=1.37)	0.34
Cancer	*n*=21; *M*=3.60 (*SD*=1.47)	*n*=18; *M*=3.72 (*SD*=1.29)	0.79
Epilepsy	*n*=7; *M*=4.63 (*SD*=0.73)	*n*=11; *M*=4.30 (*SD*=1.09)	0.49
HIV	*n*=9; *M*=2.80 (*SD*=1.75)	*n*=0	—
Glaucoma	*n*=2; *M*=1.50 (*SD*=2.26)	*n*=0	—
IBS	*n*=88; *M*=3.53 (*SD*=1.31)	*n*=115; *M*=3.45 (*SD*=1.34)	0.67
Colitis/Crohn's disease	*n*=23; *M*=4.28 (*SD*=0.91)	*n*=20; *M*=4.09 (*SD*=1.09)	0.52
Tremor	*n*=16; *M*=3.45 (*SD*=1.87)	*n*=15; *M*=3.72 (*SD*=1.25)	0.64
Anxiety	*n*=385; *M*=3.48 (*SD*=1.37)	*n*=423; *M*=3.57 (*SD*=1.32)	0.37
Depression	*n*=369; *M*=3.84 (*SD*=1.27)	*n*=330; *M*=3.67 (*SD*=1.28)	0.08
Spasticity	*n*=125; *M*=3.57 (*SD*=1.24)	*n*=132; *M*=3.58 (*SD*=1.19)	0.95
Nausea	*n*=157; *M*=4.24 (*SD*=1.03)	*n*=223; *M*=4.20 (*SD*=0.98)	0.68
Pain	*n*=450; *M*=3.46 (*SD*=1.18)	*n*=406; *M*=3.54 (*SD*=1.11)	0.28
Tics	*n*=22; *M*=3.75 (*SD*=1.46)	*n*=14; *M*=3.51 (*SD*=1.39)	0.64
Appetite	*n*=60; *M*=4.40 (*SD*=0.86)	*n*=80; *M*=4.18 (*SD*=1.05)	0.20
Seizures/spasticity	*n*=21; *M*=4.41 (*SD*=0.87)	*n*=14; *M*=3.66 (*SD*=1.00)	0.02
Headaches/migraines	*n*=206; *M*=3.79 (*SD*=1.24)	*n*=289; *M*=3.47 (*SD*=1.23)	**0.004**

## Discussion

The purpose of the present study was to examine sex differences in cannabis use and effects using a large sample of recreational and medical cannabis users. Overall, the results indicated that there are small but significant sex differences in cannabis use patterns, acute effects, and withdrawal effects. In contrast, only trivial sex differences in medical cannabis use, and in perceived efficacy of medical cannabis, were detected.

Consistent with previous research our findings indicate that men use cannabis more frequently and in higher quantities than do women. We did not replicate previous findings that men are more likely to initiate use at a younger age.^18–20^ This may be indicative of recent changes in the socio-cultural perception of cannabis use among female adolescents. However, our results do provide evidence that women are twice as likely as men to initiate use after the age of 30. This may reflect new trends in diminishing stigma toward cannabis use and decreases in older women's perceptions of risks associated with cannabis use. Alternatively, it may reflect our finding of a higher percentage of women than men reporting using cannabis for medical purposes and a lower percentage of women than men reporting using cannabis for recreational purposes.

The present study provides new insights into sex differences in the methods used to administer cannabis. Specifically, men were more likely than women to report primarily smoking joints/blunts and using concentrates and vaporizers, whereas women are more likely than men to report primarily using pipes and oral methods of administration (edibles, tinctures, capsules). Joints/blunts and some concentrates require more skill to prepare, while pipes, and oral ingestion are easier and more discrete methods. As such, sex differences in methods of administration may reflect women's tendency to be less experienced and/or more discrete about their cannabis use.

Findings from the present study also revealed a number of sex differences in the acute effects of cannabis intoxication. Somewhat surprisingly, men were more likely than women to report memory improvement when high. This result is contradictory to previous controlled laboratory research that indicates that an acute dose of sublingually administered THC improves the spatial working memory performance of female, but not male, cannabis users.^42^ Therefore, it is possible that our effect is biased by the self-report nature of our survey. In other words, men may simply be more likely to perceive and report these experiences than women. Similarly, previous research suggests that cannabis users may be more susceptible to false memories/memory distortions,^50^ leaving open the possibility that part of the experienced improvement involves the recall of false, rather than veridical, memories. To our knowledge no research has examined potential sex differences in false memories of cannabis users, but such research may prove to be enlightening. Further, our study did not distinguish between the many different types of memory (e.g., implicit, explicit, verbal, spatial) and as such future research should include more fine-grained analyses into the various types of memory impairments and improvements that men and women report experiencing when under the influence of cannabis.

Men in the present study were also significantly more likely to report experiencing altered time perception, increased musicality, and enthusiasm when high. These findings are also novel and somewhat surprising. While it is known that acute cannabis intoxication alters time perception,^51^ to our knowledge, sex differences in this effect have not been previously discovered. Future research employing time estimation tasks is needed to examine this effect objectively. Finally, our finding that women were more likely than men to report a desire to clean likely reflects the fact that women are more likely to be responsible for cleaning duties. It is also possible that some women interpreted this item to include personal cleaning and self-care, which in general women are also likely to perform more frequently.

It is widely known that acute cannabis intoxication results in increased appetite. Our results further reveal that a larger percentage of men than women reported increased appetite, while a larger percentage of women than men reported decreased appetite when high. These sex differences are consistent with the results of animal research showing that a cannabinoid agonist increased the consumption of sweetened condensed milk in male rats in the first, second, and third hour post-injection, while it only increased consumption in the female rats in the third hour post-injection.^52^ Similar sex differences in cannabinoid-induced hyperphagia have been reported in guinea pigs.^53^ Finally, these findings are consistent with research showing that an acute dose of the cannabinoid agonist dronabinol resulted in a higher fasting gastric volume (associated with reduced satiation) in men and slowed gastric emptying (associated with decreased appetite) in women.^54^ Future research may consider examining the types of cravings (e.g., sweets, carbohydrates) that cannabis users experience and whether they vary as a function of sex.

Sex differences in the specific withdrawal symptoms reported by men and women were also detected. For instance, men were more likely than women to report experiencing sleep disturbances and vivid dreams during withdrawal. This result is consistent with previous research showing that men abstaining from cannabis spend less time sleeping and less time in rapid eye movement (REM) sleep than women abstaining from cannabis.^55^ Dream intensity increases with REM deprivation^56^; therefore, men's more frequently experience of vivid dreams may be a function of their decreased time in REM sleep during withdrawal.

Our findings indicate that women are more likely than men to report experiencing nausea and anxiety during withdrawal. Consistent with these effects female medical cannabis users were more likely than male medical cannabis users to report using cannabis to treat nausea and anxiety. However no sex differences in the perceived efficacy of cannabis in treating nausea or anxiety were detected. Likewise, female medical cannabis users were more likely than male medical cannabis users to report using cannabis to treat anorexia, IBS, and headaches/migraines. However, no sex differences in the perceived efficacy of cannabis in treating anorexia or IBS were detected, and *men* reported that cannabis is more effective in treating headaches/migraines. These patterns of results likely reflect women's higher base rates and increased susceptibility to experience and report nausea,^57–59^ anxiety,^60^ anorexia,^60^ IBS,^61^ and headaches/migraines^62–64^ rather than cannabis-specific effects.

As with most survey studies, the present study is limited by potential bias in self-reports, including but not limited to, exaggeration of beneficial effects, diminishment of negative effects, recall bias, and in the case of the medical data potential placebo effects. An additional limitation concerns our findings of sex differences in frequency, quantity, and method of use and the impact that these differences may have on our findings of acute and withdrawal effects. It is possible that men's increased frequency and quantity of use and their preference for different routes of administration may be impacting or driving some of the sex differences in the acute and withdrawal effects. For instance, it is possible that we did not replicate previous findings of increased withdrawal symptoms in women^32,33^ because of their lower frequency and quantity of use. Future more controlled research will need to examine these possibilities and further validate our results.

While our findings provide intriguing insights into sex differences in the use and effects of cannabis, caution should be taken when generalizing results from this self-selected convenience sample to the general population. Our exclusion of individuals who had not used cannabis in the past 90 days was practical and ensured that the survey questions would be relevant to participants; however, it limits the generalization of our findings to recent cannabis users. The sample was also predominantly Caucasian and lacked ethnic diversity. It is possible that individuals from minority groups, who are more likely to be arrested for cannabis-related offenses,^65,66^ were more hesitant to participate. While individuals from a variety of different countries and regions completed the survey, the origin of the survey was Washington State, and the majority of participants were from this state. Regional differences in the availability of different forms of cannabis and in medical cannabis laws may have therefore affected some of the outcomes. Despite our use of a convenience sample of predominantly Caucasian, recent cannabis users residing in Washington State, our sample was very large and well balanced with respect to sex, which increases our confidence in the generalizability of the present findings to this specific population of cannabis users.

These results can be used to guide future research on biological and psychosocial mechanisms underlying cannabis-related sex differences and to inform clinicians treating individuals with cannabis use disorders. Our findings indicate that men attempting to quit cannabis may benefit from sleep aids, while women may benefit from anti-emetics and training in anxiety coping strategies. Finally, the results should be used to inform the growing population of cannabis consumers, policymakers, and medical cannabis service providers, about some of the risks and benefits of cannabis for both sexes so they can make more informed decisions about the use and regulation of cannabis.

## Abbreviations Used

IBS: irritable bowel syndrome
n/a: not applicable
REM: rapid eye movement
THC: tetrahydrocannabinol
